# Assessment of Exposure to Aluminum through Consumption of Noodle Products

**DOI:** 10.3390/foods12213960

**Published:** 2023-10-30

**Authors:** Nalinrat Kongta, Kunchit Judprasong, Rodjana Chunhabundit, Jintana Sirivarasai, Weeraya Karnpanit

**Affiliations:** 1 Master of Science Program in Nutrition, Faculty of Medicine, Ramathibodi Hospital and Institute of Nutrition, Mahidol University, Bangkok 10400, Thailand; nalint.may@gmail.com; 2 Institute of Nutrition, Mahidol University, Salaya, Nakhon Pathom 73170, Thailand; 3 Nutrition Division, Faculty of Medicine Ramathibodi Hospital, Mahidol University, Bangkok 10400, Thailand; rodjana.chu@mahidol.ac.th (R.C.); jintana.sir@mahidol.ac.th (J.S.); 4 School of Science, Western Sydney University, Locked Bag 1797, Penrith, NSW 2751, Australia

**Keywords:** aluminum, risk assessment, noodle products, Al-containing food additives

## Abstract

This study aimed to determine aluminum (Al) contents in commonly consumed noodles and estimate the risk of Al exposure through the consumption of noodles in the Thai population. A total of 80 samples, 20 samples each of rice stick noodles, egg noodles, wide rice noodles, and Thai rice noodles was purchased from markets in Bangkok Metropolitan and other four provinces in each region of Thailand. Wet digestion and graphite furnace atomic absorption spectrometry (GFAAS) were used to determine Al contents. Exposure assessment of Al was conducted by applying the consumption amounts of noodles from the national consumption survey and the Al contents of the noodle samples. The hazard quotient (HQ) was calculated to estimate the risk of exposure to Al. Aluminum contents in the noodles ranged from not detected to 630 mg/kg. Exposure to Al from consumption of each noodle product in all age groups was lower than the provisional tolerable weekly intake (PTWI). However, Al exposures in children calculated from the high consumer models and Al exposures in all age groups from the worst-case scenarios were higher than the PTWI, indicating potential adverse health effects. Consumers who regularly consume large amounts of noodle products containing Al may be at risk of having adverse health effects.

## 1. Introduction

Aluminum (Al) is an ubiquitous element distributed throughout the environment. Humans may be exposed to Al in their daily lives via different sources, including water, drugs, vaccines, cosmetics, and food [1]. Foodstuffs, especially those containing Al-containing additives, are the major source of Al intake [2]. In modern mass food production, food additives play a crucial role in preserving the nutritional value of foods, enhancing and sustaining food quality throughout production, processing, packaging, transportation, and storage. Aluminum-containing food additives have long been utilized in food processing as firming agents, raising agents, stabilizers, anti-caking agents, and coloring agents. Aluminum ammonium sulfate (INS no. 523), sodium aluminum phosphates (INS no. 541(i)), and sodium alumino silicate (INS no. 554) are among the Al-containing food additives listed in the Codex General Standard for Food Additives [3]. These additives are permitted to be added in a variety of food products including batters, flours, noodles, and pasta [3]. People may consume a variety of foods with Al-containing food additives and may unintentionally intake a high level of Al exceeding the acceptable intake.

An excessive amount of Al intake can pose adverse health effects. Although Al is widely found in food, water, and some medicines, there have been no reports of acute toxicity from Al intake in the general population [4]. Toxicity of Al includes damage to the hematological system, bone marrow, and brain by causing cell death as a result of increased oxidative stress and lipid peroxidation [4,5]. Aluminum is not classified as a mutagen and carcinogen in animals, and there is insufficient data on Al carcinogenicity in humans [6]. Previous studies in animals revealed that soluble Al compounds are toxic to the development and reproduction systems. After being exposed to aluminum via dialysis fluids, individuals with chronic renal failure, as well as adults who have been consuming aluminum-containing antacids daily for extended periods, have both been documented to develop osteomalacia [4]. The neurotoxicity of Al has received attention in recent decades due to the assumption that it is linked to Alzheimer’s disease. Increased levels of Al have been detected in the brains of individuals with Alzheimer’s disease. However, it remains uncertain whether this is a contributing factor to the condition or a consequence of it [7]. This uncertainty likely arises from the complex and highly variable nature of the disease presentation [8]. The Joint FAO/WHO Expert Committee on Food Additives (JECFA) established a provisional tolerable weekly intake (PTWI) of 2 mg/kg body weight (bw)/week for Al [9], while the European Food Safety Authority (EFSA) set the tolerable weekly intake (TWI) at 1 mg/kg bw/week [10].

High levels of Al exposure have been reported in several countries, especially among vulnerable groups such as children. A previous study on dietary Al exposure in Shenzhen, China, revealed that children under 14 years old had high Al intake levels, exceeding the JECFA PTWI for Al [11]. The primary contributors to Al intake included fried twisted crullers, leafy vegetables, and bean products [11]. Another study indicated that wheat flour and puffed products were the primary sources of dietary Al exposure, with children being more susceptible to elevated Al exposure [12]. A previous study conducted a risk assessment of Al exposure through the consumption of infant formulas and baby biscuits in infants and toddlers. The study reported high target hazard quotient (HQ) values ranging from 10 to 20, calculated by dividing the estimated daily intake by the oral reference dose of aluminum phosphide (4 × 10^−4^ mg/kg bw/day). Hazard quotient values greater than one indicate the potential for adverse effects in the studied population [13]. Tajdar-oranj et al. conducted a risk assessment of Al exposure from noodle consumption in Iranian children and adults, which indicated that regular consumption of instant noodles can lead to certain adverse health effects associated with Al exposure [14].

Wheat flour and wheat-based foods, such as deep-fried dough sticks, steamed bread, pastries, and noodles, are known to contain high levels of Al [15,16]. Noodles are a dietary staple in Thailand, and a variety of noodle products made from rice flour and wheat flour are available in the markets. According to Thailand’s food standards, the use of Al-containing food additives is permitted in flour and certain noodle products [17]. Therefore, if Al-containing additives are used in noodle production, they may contain higher levels of Al compared to those without such additives. A study reported elevated levels of Al in hair samples collected from Thai children aged 3–7 years, living in Bangkok and nearby suburbs. Approximately 12% (13 out of 111 subjects) had hair Al levels equal to or exceeding the reference limit of 22 µg/g [18]. The researchers hypothesized that the high Al exposure observed in some participants could be attributed to regular consumption of Al-contaminated foods and/or the use of Al-containing adjuvants in vaccines [18]. Published information regarding Al content and Al intake from the consumption of noodle products containing Al-containing food additives among the Thai population is limited. This study aimed to determine the Al content in commonly consumed noodles and to estimate Al dietary intake, along with potential health risks in the Thai population.

## 2. Materials and Methods

### 2.1. Chemicals

The aluminum standard for AAS (TraceCERT^®^ 1000 mg/L in nitric acid) was purchased from Sigma-Aldrich (St. Louis, MO, USA). Nitric acid (EMSURE^®^ 65%) was purchased from Merck (Darmstadt, Germany).

### 2.2. Noodle Samples

The sample size was calculated using G*power program (version 3.1.9.2, Düsseldorf, Germany) in accordance with one-way ANOVA, considering an effect size of 0.40, α level of significance of 0.05, a desired power of 0.8, and 4 groups of noodles (Thai rice noodles, wide rice noodles, rice stick noodles, and egg noodles). The resulting sample size was 76 noodle samples in total. A total of 80 noodle samples was randomly collected from markets, comprising 20 samples in each group. The study employed a multi-stage stratified sampling design to gather samples, accounting for the population numbers in each individual province. The representative provinces were selected from the northern, eastern, northeastern, and southern parts of Thailand, specifically Chiang Mai, Chonburi, Nakhon Ratchasima, and Nakhon Si Thammarat, respectively. From the total number of 20 samples of each noodle type, the ratio of the population in the five provinces was applied to calculate the number of samples to be collected from each province. The number of samples collected from Bangkok, Nakhon Ratchasima (northeastern), and Chiang Mai (northern) was 9, 4, and 3 samples, respectively. Two samples of each noodle type were collected from Chonburi (eastern) and Nakhon Si Thammarat (southern).

### 2.3. Method Validation

The method performance characteristics that examined the validity of the Al analysis in this study included working range, linearity, instrumental/method detection and quantification limits, accuracy, precision, and interlaboratory comparison. Method validation was conducted according to the Eurachem Guide [19]. To confirm the suitability of the analytical method, the results of the characteristic checks were compared with the acceptance criteria [20]. For validation of the working range and linearity, seven concentrations of Al solutions of 10, 20, 30, 40, 60, 80, and 100 μg/L were prepared by serial dilution from a stock standard Al solution (1000 mg/L). A standard curve of Al was plotted between Al concentration (x axis) and absorbance (y axis). The linear correlation of the measured concentration range was evaluated using the coefficient of determination (R^2^), of which the acceptable linearity criteria is R^2^ = 0.995 [20]. Instrumental LOD and LOQ were verified by determination of reagent blank solutions (3% nitric acid), while procedural blank solutions were prepared and analyzed for Al to estimate the LOD and LOQ of the analytical method. The LOD and LOQ were calculated using the standard deviation (S_0_) of the reagent blank and sample blank analysis using Equation (1):

(1) $${S_{0}}^{\prime }\;=\;S_{0}/\sqrt{n}$$

where *n* = 3 (number of replications). LOD = 3S_0_′. LOQ = 10S_0_′.

Trueness of Al determination was studied using the spiking method. The Al standard solutions of three concentrations—low (10 μg/L), medium (50 μg/L), and high (100 μg/L)—were spiked to the sample solutions and further measured for Al concentration. Ten replicates of each Al concentration were determined. Recovery rate was calculated by determining the percentage of the measured spike in the matrix sample compared to the amount of spike added to the sample using Equation (2):

%Recovery = (Al in spiked sample − Al in non-spiked sample)/actual spiked Al(2)

The repeatability of the analytical method for Al was determined. The mean and standard deviation (SD) of Al concentration were calculated. The relative standard deviation of repeatability (RSD_r_) and the predicted relative standard deviation of repeatability (pRSD_r_) values were calculated using Equations (3) and (4), respectively:

RSD_r_ = SD_r_ × 100/mean (3)

pRSD_r_ = 0.66 × 2C^−0.1505^ (4)

The HorRat value was calculated using Equation (5):

HorRat = RSD_r_/pRSD_r_ (5)

HorRat values calculated from various concentrations were compared with the AOAC acceptance criteria [20].

Interlaboratory comparison of Al analysis between the results from this study and a laboratory with ISO/IEC 17025 [21] accreditation (SGS Thailand Limited, Bangkok, Thailand) was performed. The benchmark laboratory determined Al in foods using the in-house method based on AOAC (2019) [20] method number 999.10 and 2011.14. Three samples of noodle products; rice stick noodles, wide rice noodles, and Thai rice noodles, were selected for the interlaboratory comparison. The paired *t*-test statistic was used to determine the difference in Al concentrations.

### 2.4. Determination of Al Contents in Noodles

The aluminum contents in the noodle samples were analyzed based on the study of Saiyed and Yokel [22] with some modifications. Noodle samples were homogenized with deionized water in a 1:1 ratio (*w*/*w*), and the closed-vessel thermal heating wet digestion technique using concentrated nitric acid was employed to decompose organic matter. The digested solution was diluted with deionized water and the Al concentration determined using GFAAS (PinAAcle 900Z, PerkinElmer Inc., Shelton, CT, USA). The operating parameters and furnace conditions of the GFAAS for the Al analysis are shown in Table 1 and Table 2, respectively.

The moisture contents of the homogeneous noodle samples were determined based on the AOAC 945.15 method [20]. Moisture contents were used to convert Al concentration from wet weight basis to dry weight basis.

### 2.5. Effect of Cooking

Since rice stick noodles and egg noodles are typically consumed as boiled noodles, there is a possibility of aluminum content leaching into the boiling water. This study investigated the effect of cooking on Al concentration in rice stick noodles and egg noodles. The method of cooking of rice stick noodles followed a method of Surojanametakul et al. [23]. The rice stick noodles were boiled with 150 mL deionized water for 10 min, rinsed with 20 mL deionized water, and put into a strainer. The egg noodles were boiled with 500 mL of deionized water for 9 min and immediately chilled in 200 mL of deionized water. The cooking process was not investigated for Thai rice noodles and rice thick noodles, as they are typically consumed fresh.

### 2.6. Consumption Data of Noodles

Data on the consumption of noodles by the Thai population were sourced from the 2016 food consumption survey conducted by the National Bureau of Agricultural Commodity and Food Standards, Ministry of Agriculture, and Cooperatives in Thailand [24]. The dataset encompassed 8478 participants, spanning various age groups, including those under 3 years old and those over 3 years old. These participants were selected from both Bangkok and the four other regions of Thailand, namely North, Northeast, Central, and South. The obtained consumption data are presented as the mean and the 97.5th percentile consumption per capita and for individuals who consume the product (eater only).

### 2.7. Exposure Assessment

A deterministic estimation of exposure to Al from the consumption of noodle products by the Thai population was conducted in accordance with the WHO guidelines [25]. The unit of Al exposure was expressed as mg/kg bw/week. Various exposure scenarios were considered, including the average and 97.5th percentile (PCTL) consumption of noodle products, as well as the average and 97.5th PCTL of Al concentration. The estimated daily intake scenario was calculated using the mean consumption of noodles (per capita) and the mean and median Al concentration. The high consumer scenario was calculated using the 97.5th PCTL consumption of noodles (eater only) and the mean and median Al concentration. The worst-case scenario was calculated using the 97.5th PCTL consumption of noodles (eater only) and the 97.5th PCTL Al concentration. A lower-bound scenario was performed by replacing Al levels below the LOD or LOQ with a value of zero. Equation (6) was used to evaluate weekly exposure to Al from noodle consumption:

(6) $$Al\;exposure=\frac{concentration\;of\;Al\times consumption\;amount\;of\;noodles\times 7}{body\;weight}$$

where unit of Al exposure = mg/kg bw/week, concentration of Al = mg/kg, consumption amount = kg/day, body weight = kg, 7 = conversion factor of day to week.

### 2.8. Risk Characterization

The risk characterization of Al exposure from noodle consumption was estimated by using the HQ, which compares the Al exposure with the PTWI of Al established by JECFA [9] and EFSA [10]. If the HQ is lower than or equal to 1, it implies that there is no risk of adverse health effects from exposure to Al through the consumption of noodles. However, if the HQ is greater than 1, it implies that there may be adverse health effects from exposure to Al through the consumption of noodles [24]. The risk of exposure to Al as HQ from the consumption of noodles was calculated using Equation (7):

HQ = Al exposure/PTWI of Al(7)

### 2.9. Statistical Analysis

The levels of Al in the noodle products were presented as mean ± standard deviation, median, and 97.5th PCTL. The normal distribution of data was examined using the Kolmogorov–Smirnov test. For non-parametric distribution, the Kruskal–Wallis test and Mann–Whitney U-test were used to determine the statistical significance of Al concentrations among different noodle products. A probability level of *p* < 0.05 was considered statistically significant. The statistical tests were conducted using the SPSS Statistical Analysis Software Program, version 19 (IBM Corporation, Armonk, NY, USA).

## 3. Results and Discussion

### 3.1. Method Validation of Determination of Al in Noodles

Regarding the working range and linearity characteristics, the coefficient of determination (R^2^) for the calibration curves was greater than 0.995. This indicates that the calibration curves for the working Al standard with concentrations of 10, 20, 30, 40, 60, 80, and 100 µg/L were fitted with a linear regression line and suitable for analysis. The instrumental LOD and LOQ for Al analysis using GFAAS were 1.53 and 5.09 µg/L, respectively. The LOD and LOQ for the analysis of Al content in the noodle products in this study were 1.95 and 6.51 µg/kg, respectively. In terms of accuracy, the percentage recoveries from spiked samples with standard Al concentrations of 10, 50, and 100 µg/L were within the range of 87.19% to 108.30%. The Al analysis procedure employed in this study met the AOAC acceptance criteria [20] for percentage recoveries (80–110%) at all three concentrations. Regarding the precision of the analytical method for Al determination, the RSD_r_ values for Al at low, medium, and high concentrations were 6.55%, 4.75%, and 3.45%, respectively. When assessing precision using the Horwitz ratio according to the AOAC criteria [20], the HorRat values for the analyses of aluminum at low, medium, and high concentrations were 0.31, 0.28, and 0.23, respectively. The AOAC has established a HorRat acceptance criterion of ≤2 [20]. It was determined that the Al analysis method employed in this study satisfied the criteria for repeatability. In the interlaboratory comparison of Al analysis, there was no significant difference (*p* > 0.05) in Al contents of the noodle samples between the results obtained from this study and the laboratory with ISO/IEC 17025 [21] accreditation.

Atomic absorption spectroscopy, particularly GFAAS, is a non-flammable atomization method frequently used for Al determination. GFAAS offers higher sensitivity compared to flame-AAS and ICP-OES [26]. Despite the growing popularity of ICP-MS for metal analysis, including Al, it is better suited for simultaneous analysis of multiple heavy metals and involves expensive instruments and highly skilled analysts. Therefore, employing GFAAS for Al analysis in noodle products in this study was a suitable and cost-effective approach. In the method validation for Al analysis in noodle products, R^2^ values of greater than 0.995 were observed within the 10–100 µg/L working range, indicating a linear relationship of the calibration curve. Accuracy and precision tests complied with AOAC acceptance criteria through percent recovery and HorRat calculations [20]. As per the Codex Alimentarius Commission, the chemical analysis method must achieve LOD and LOQ values less than or equal to 10 and 5 times the maximum level (ML), respectively [27]. Considering a maximum level of Al of 300 mg/kg in the particular noodle product [17,28], the analysis method should ensure that the LOD and LOQ values remain at or below 30 and 60 mg/kg, respectively. The LOD and LOQ values achieved in this study are consistent with the target concentrations.

### 3.2. Concentration of Al in the Noodle Product Samples

The aluminum concentrations, presented as mg/kg wet weight (ww) and mg/kg dry weight (dw), are shown in Table 3. The rice stick noodles had the highest mean and median Al concentrations both on a wet weight and dry weight basis, followed by wide rice noodles. Significant differences in Al content were observed among the different types of noodle products. Additionally, the wide rice noodle samples had significantly (*p* < 0.05) higher Al levels compared to Thai rice noodle samples. The percentages of noodle products that did not comply with Codex GSFA and Thailand’s food standards regarding aluminum-containing food additives were found to be 95% for rice stick noodles, 55% for wide rice noodles, and 30% for Thai rice noodles. However, no detectable Al concentration was found in any of the egg noodle samples.

In accordance with the Codex GSFA and the Notification No. 418 of the Ministry of Public Health in Thailand, sodium aluminum phosphates (ML = 1600 mg/kg) are permitted in food category number 6.2.1 (flours) [17,28]. However, this allowance applies solely to self-rising flour, with regular wheat flour being excluded [17,28]. Aluminum ammonium sulfate (ML = 300 mg/kg) is permitted in food category number 6.4.1 (fresh pasta, noodles, and similar products), but only for Kuzukiri and Harusame noodles [17,28]. These noodles, originating from Japan, are made from Kuzu root and mung bean starch, respectively. Therefore, aluminum-containing food additives are not permitted in rice stick noodles, egg noodles, wide rice noodles, and Thai rice noodles. However, high detection rates were found in the three rice noodle products. Aluminum was not only found in unbranded noodles; high aluminum concentrations were also detected in samples with a brand name, place of production, or an FDA food serial number on their labels. It is crucial to educate and communicate food additive regulations to food producers. Furthermore, food safety authorities in Thailand should conduct regular monitoring and surveillance concerning food additives.

Table 4 presents concentrations of Al in noodles and similar products in this study and previous studies [14,15,29,30,31,32]. Depending on the type of noodle products, the concentration of aluminum in the noodles in this study was either similar to that in some previous studies or higher than in others. In our study, Al was not detected in any of the egg noodle samples, which contrasts with the Al contents found in wheat noodles as reported in the study of Ma et al. [15]. A previous study indicated that Al contents in noodles varied significantly among different companies [29].

Traditionally, to improve the textural properties of noodles and reduce cooking loss, it has been recommended to include alum (aluminum ammonium sulfate or potassium aluminum sulfate) during production [33]. Alum is a frequently used additive in cereal flour and starch-based products such as noodles [33]. The inclusion of alum could enhance the quality of potato noodles by improving their elasticity, tensile strength, and sensory attributes [33]. Nevertheless, with growing concerns about potential adverse health effects linked to Al residue from alum usage, several researchers have explored the use of alum substitutes and identified suitable alternatives such as calcium carbonate, calcium silicate, potassium alginate, and potassium dihydrogen phosphate [33,34].

### 3.3. Effect of Cooking on Al Contents in Noodle Products

The effect of boiling on Al content was only studied in the rice stick noodle samples, as none of the egg noodle samples contained Al. It was observed that the concentration of Al in cooked rice stick noodles was approximately 3.7 times lower than that of raw samples, resulting in around a 72% reduction in Al content after cooking. However, rice stick noodles are commonly consumed both in soup, where they are boiled in hot water and the boiling water is discarded, and in stir-fried dishes without prior boiling.

According to a study by Lee et al. [35], it was demonstrated that noodles contained 15.36 mg of Al per one kg of noodles. After boiling for 3, 5, and 10 min, the Al concentrations decreased to 5.14, 4.34, and 4.06 mg/kg, respectively. Over a 10 min boiling period, the levels of Al significantly decreased (*p* < 0.05) by 74% [35]. As boiling time increased, the Al content decreased significantly. Consistent with the previous finding, it was observed that boiling rice stick noodles reduced Al levels by 72% (on a wet weight basis). Al has the ability to leach and dissolve in water during the boiling process [35]. Therefore, noodles are less likely to contain Al if they are consumed after boiling and the cooking water is drained.

### 3.4. Consumption Data of Noodles

The results of consumption data of noodles product by age group in the Thai population are shown in Table 5. Consumption of rice stick noodles, wide rice noodles, and Thai rice noodles as per capita and eater only at the mean and 97.5th PCTL levels were applied for the exposure assessment. In all age groups, the highest mean (per capita) and 97.5th PCTL (eater only) consumption were found from Thai rice noodles at 23.66 and 512 g/person/day respectively. The highest consumption amount (g/person/day) of all noodle products was found in the 18–34.9 years. The average body weights of the Thai population in the age groups of 3–5.9, 6–12.9, 13–17.9, 18–34.9, 35–64.9, and ≥65 years old were 17.25, 33.38, 53.42, 63.12, 63.53, and 55.77 kg, respectively. These weights were used for the calculation of Al exposure per kilogram of body weight.

### 3.5. Dietary Exposure Assessment and Risk Characterization of Al from Consumption of the Noodle Products

**Figure 1:**
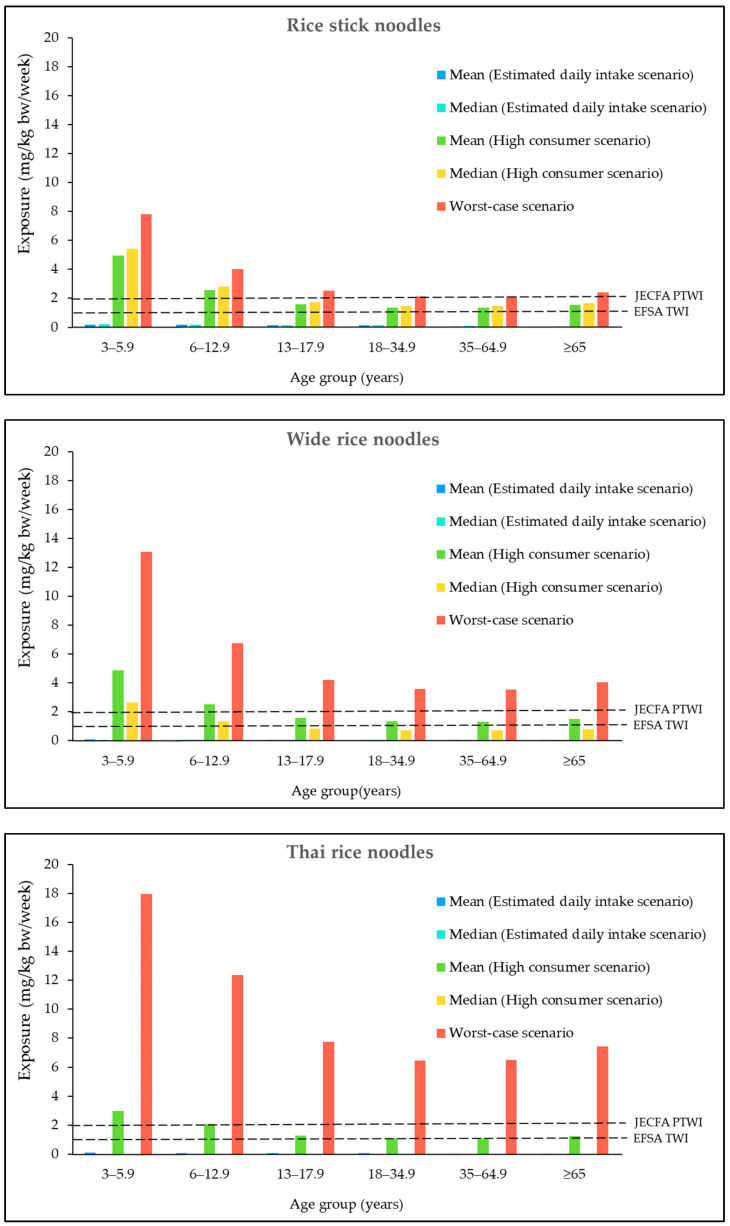
Exposure to Al (mg/kg bw/week) from the consumption of noodle products, specifically rice stick noodles, wide rice noodles, and Thai rice noodles, in the Thai population. Al exposure from the consumption of egg noodles was not calculated as Al was not detected in any of the egg noodle samples. JECFA PTWI: Provisional tolerable weekly intake of 2 mg/kg bw/week established by the Joint FAO/WHO Expert Committee on Food Additives. EFSA TWI: tolerable weekly intake of 1 mg/kg bw/week established by the European Food Safety Authority.

Figure 1 presents the Al exposure from the consumption of noodle products in each individual age group. Among all age groups, the highest Al exposure from the consumption of each type of noodle, in all exposure scenarios, was found in children aged 3–5.9 years. In both the estimated daily intake and high consumer scenarios, children aged 3–5.9 years had the highest exposure to Al from the consumption of rice stick noodles. However, in the worst-case scenarios, the highest Al exposure was found from the consumption of Thai rice noodles.

The health risk of Al exposure from noodle consumption was calculated as HQ (Table 6). The HQ values obtained by dividing the Al exposure by the PTWI were established by JECFA (2 mg/kg bw/week) or EFSA (1 mg/kg bw/week). In comparison to the JECFA PTWI for Al, the average consumption of individual noodle products containing the mean or median Al levels may not result in adverse effects. High consumption of noodle products containing the mean Al levels could potentially lead to adverse health effects in children’s groups. In the worst-case scenario, the HQ values for Al exposure consumption of individual noodle products exceeded 1 across all age groups. Based on the HQ calculation using the EFSA TWI, the consumption of individual noodle products containing the mean or median level of Al may not lead to adverse health effects. However, the high consumption of individual noodle products containing the mean Al level could potentially result in adverse health effects across all age groups. In the worst-case scenario, the HQ values for Al exposure from consumption of any noodle products were greater than 1 across all age groups.

Several previous studies have specifically reported Al exposure from consumption of noodles or similar products. In the study by Ma et al. [30], when comparing Al exposure with the JECFA PTWI, the HQ for average Al exposure through noodle consumption ranged from 0.1 to 0.16, while the 95th PCTL exposure ranged between 0.06 to 0.44 across the six age groups studied [30]. The previous study indicated that exposure to Al from noodle consumption alone may not pose a health risk. However, similar to our study, it was found that exposure to Al from noodle consumption, especially in high-consumption scenarios, could exceed the health-based guidance value (HBGV). The study by Tajdar-Oranj et al. [14] reported HQ values exceeding 1 for the 95th PCTL of Al exposure from instant noodle consumption in children and adults, with values of 6.2 and 1.8, respectively. These findings suggest a potential health risk [14]. Zhang et al. [1] reported that average dietary intake of Al from noodles was 0.133 mg/kg bw/week (6.65% of PTWI) and that of 97.5th PCTL was up to 0.859 mg/kg bw/week (42.95% of JECFA PTWI). While children under the age of 14, those living in Northern China, and consumers with high consumption levels had Al exposure of higher than the PTWI [36]. Stahl et al. reported that Al exposures from consumption of pasta in German children and adults were 23 and 10% of EFSA TWI, respectively [32].

Previous studies conducted dietary Al exposure from consumption of various food stuffs. Ma et al. studied Al exposure using consumption data from the Nutrition and Health Survey of Chinese Residents and the Nutrition and Health Monitoring of Chinese Residents; the study reported that average dietary intake of Al was at 1.80 mg/kg bw/week which was 89.75% of JECFA PTWI [15], while high dietary exposures at the 90th and 97.5th PCTL were 4.65 and 7.66 mg/kg bw/week which were 233 and 383% of PTWI, respectively [15]. The dietary weekly aluminum (Al) intake of the Lebanese population aged 18–64 years was studied using a self-reported food frequency questionnaire. It was reported that the mean Al exposure for females and males in Lebanon was 0.49 and 0.51 mg/kg bw/week, respectively, which did not exceed the EFSA TWI for Al [37]. Probabilistic dietary exposure to Al in the Taiwanese population (0–65+ years old) using the Taiwan National Food Consumption Database was studied [38]. Cake, waffle, kelp, snacks, and bread were the major contributors of Al exposure. The study found that high dietary exposure at the 75th PCTL of Al for Taiwanese children exceeded the JECFA PTWI, indicating a potential adverse health effect [38]. Dietary intakes of Al in Irish children and adults were calculated based on the Al content per serving as specified in the manufacturer’s instructions for the food products [39]. It was reported that consuming combinations of food products in children would exceed EFSA TWI. Baby foods contained a significant amount of Al relative to their weight, and it is highly likely that they exceed the PTWI for this group, especially among customers loyal to a particular brand. Irish adults can also potentially reach the PTWI, particularly if they consume processed fruits, vegetables, and canned fish. Despite the presence of many food and beverage products with low Al content on the market, consumers can still readily exceed the PTWI by consuming a combination of such foods [39]. Previous research in Japan studied the Al intake from consumption of foods with Al-containing food additives. The study revealed that 21% of the studied food products had Al levels exceeding the EFSA TWI for young children when they consumed two servings per week [40].

Apart from diet, people can be exposed to Al from other sources, including drinking water, cosmetics, personal care products, medications, and food contact materials [41,42]. Depending on the levels of Al contained in the water source and whether Al flocculants are used during water treatment, varying levels of Al can be detected in drinking water [2]. Nevertheless, the excessive use of Al salts, such as alum, and Al polymers, such as poly-aluminum chloride, in the coagulation and flocculation processes of water treatment raises concerns about increased Al levels in drinking water [43,44]. Aluminum is present in numerous formulations of cosmetics and personal care items, including antiperspirants, lipsticks, liquid makeup foundations, and toothpaste [45]. Aluminum salts, particularly aluminum chlorohydrate, were frequently applied in personal care items like antiperspirants for the purpose of managing perspiration [46]. However, people are exposed to aluminum from cosmetics and personal care products to a lesser extent than from dietary sources [45]. Medications constitute a significant contributor to Al intake, with the most noteworthy sources being medications that contain Al, including antacids, antidiarrheal drugs, buffered aspirins, and vaccine adjuvant [46,47]. Human exposure to a significant amount of Al can occur through the use of Al cookware, Al foil for food packaging, and the consumption of food stored in Al cans [41]. Aluminum has the potential to transfer from cookware into food, and this transfer becomes more pronounced with higher food acidity, elevated temperatures, and prolonged exposure [41].

## 4. Conclusions

Four commonly consumed noodles (80 samples) were collected from Bangkok and four other provinces in Thailand. Almost all the rice stick noodles, half of the wide rice noodles, and one-third of the Thai rice noodles contained Al levels that did not comply with the national and international food standards. Aluminum was not detected in any of the collected egg noodles. The mean weekly Al intake of the Thai population did not exceed the PTWI established by EFSA and JECFA, indicating no health risk concern. However, consumers with high noodle consumption at the 97.5th PCTL were at risk of exposure to Al through their noodle consumption. Information regarding the legal use of Al-containing food additives in noodle products should be disseminated to food producers. Food safety authorities should monitor the levels of Al in food products, and information about potential foods containing Al should be made available to consumers.

## Figures and Tables

**Table 1:** Instrumental parameters of the GFAAS for Al analysis.

Parameter	Setting
Lamp	Al Hollow cathode lamp
Lamp current (mA)	25
Wavelength (nm)	309.27
Slit width (nm)	0.7
Temperature (°C)	Pyrolysis 1200 Atomization 2300
Atomization site	Pyro/Platform
Sample volume (μL)	20
Calibration standards (µg/L)	10, 20, 30, 40, 60, 80, 100

**Table 2:** Furnace temperature program of the GFAAS for Al analysis.

Step No.	Temperature (°C)	Ramp Time (s)	Hold Time (s)	Internal Flow	Gas Type
1	110	1	30	250	Normal
2	130	15	30	250	Normal
3	1200	10	20	250	Normal
4	2300	0	5	0	Normal
5	2450	1	3	250	Normal

**Table 3:** Concentrations of Al (mg/kg ww and mg/kg dw) in the noodle product samples.

Noodle Product	Form	Concentration (mg/kg ww)			Concentration (mg/kg dw)		
		Mean ± SD	Median	97.5th PCTL	Mean ± SD	Median	97.5th PCTL
Rice stick noodles	Raw	417.96 ± 160.56	455.68 ^a^	620.68	654.70 ± 251.51	713.78 ^a^	972.24
Egg noodles	Raw	ND	ND ^c^	ND	ND	ND ^c^	ND
Wide rice noodles	Fresh	89.99 ± 98.21	48.72 ^b^	240.29	239.14 ± 261.00	129.47 ^b^	638.56
Thai rice noodles	Fresh	19.04 ± 39.02	ND ^c^	115.34	66.69 ± 136.66	ND ^c^	403.99

^a,b,c^: Different superscript letters in the same column indicated significant differences in Al content among the different types of noodle products (*p* < 0.05, Mann–Whitney U-test). mg/kg ww: milligram/kilogram wet weight. mg/kg dw: milligram/kilogram dry weight. ND: not detected (below the LOD of 1.95 µg/kg).

**Table 4:** Concentration of Al in noodle products from previous studies and this study.

Noodle Product Samples	Concentration of Al (mg/kg)	Reference
Noodles	297–767	Han et al., 1995 [29]
Remen	63–80	Han et al., 1995 [29]
Vermicelli	33–46	Han et al., 1995 [29]
Noodles	10–344	Ma et al., 2016 [15]
Noodles	1–531	Ma et al., 2019 [30]
Vermicelli	1–365	Ma et al., 2019 [30]
Steamed cold noodles	5–1127	Ma et al., 2019 [30]
Flour and noodles	3–44	Jiang et al., 2013 [31]
Vermicelli	5–395	Jiang et al., 2013 [31]
Pasta	1–76	Stahl et al., 2011 [32]
Instant noodles	7–16	Tajdar-Oranj et al., 2018 [14]
Rice stick noodles	ND–623	This study
Wide rice noodles	ND–244	This study
Thai rice noodles	ND–117	This study
Egg noodles	ND	This study

ND: not detected (below the LOD of 1.95 µg/kg).

**Table 5:** Mean and 97.5th percentile consumption of noodle products (g/person/day) as per capita and eater only.

Noodle Sample	Age (Year)	Per Capita (g/Person/Day)		Eater Only (g/Person/Day)	
		Mean	97.5th PCTL	Mean	97.5th PCTL
Rice stick noodles	3–5.9	3.98	23.14	44.66	108.00
	6–12.9	6.99	46.28	59.73	108.00
	13–17.9	9.52	54.00	64.13	108.00
	18–34.9	11.33	61.71	69.66	108.00
	35–64.9	6.19	46.29	62.53	108.00
	≥65	2.67	23.14	53.88	108.00
Wide rice noodles	3–5.9	2.66	19.14	54.22	134.00
	6–12.9	2.32	19.15	69.69	134.00
	13–17.9	3.05	38.28	81.56	134.00
	18–34.9	2.87	38.28	82.17	134.00
	35–64.9	2.16	19.15	80.25	134.00
	≥65	1.83	19.15	67.88	134.00
Thai rice noodles	3–5.9	13.49	73.16	147.03	384.00
	6–12.9	17.85	109.72	192.40	512.00
	13–17.9	20.91	109.72	226.57	512.00
	18–34.9	23.66	146.28	230.91	512.00
	35–64.9	20.42	128.00	248.92	512.00
	≥65	11.18	73.14	209.25	512.00

Source: Food Consumption Data of Thailand. The National Bureau of Agricultural Commodity and Food Standards, Ministry of Agriculture, and Cooperatives in Thailand (2016) [24]. PCTL: percentile.

**Table 6:** Risk characterization of exposure to Al from noodle consumption calculated as HQ in comparison to PTWI of JECFA and TWI of EFSA.

Noodle Product	Age	HQ Calculated from JECFA PTWI					HQ Calculated from EFSA TWI				
		Estimated Daily Intake Scenario		High Consumer Scenario		Worst-Case Scenario	Estimated Daily Intake Scenario		High Consumer Scenario		Worst-Case Scenario
		Mean	Median	Mean	Median	97.5th PCTL	Mean	Median	Mean	Median	97.5th PCTL
Rice stick noodles	3–5.9	0.091	0.099	**2.480**	**2.710**	**3.900**	0.182	0.200	**4.950**	**5.420**	**7.800**
	6–12.9	0.083	0.091	**1.280**	**1.400**	**2.020**	0.166	0.181	**2.560**	**2.800**	**4.030**
	13–17.9	0.070	0.077	0.800	0.880	**1.260**	0.141	0.154	**1.600**	**1.750**	**2.520**
	18–34.9	0.071	0.078	0.680	0.740	**1.070**	0.142	0.155	**1.350**	**1.480**	**2.130**
	35–64.9	0.038	0.042	0.670	0.740	**1.060**	0.077	0.084	**1.340**	**1.470**	**2.120**
	≥65	0.019	0.021	0.770	0.840	**1.210**	0.038	0.041	**1.530**	**1.680**	**2.410**
Wide rice noodles	3–5.9	0.048	0.026	**2.450**	**1.330**	**6.530**	0.097	0.053	**4.890**	**2.650**	**13.070**
	6–12.9	0.022	0.012	**1.260**	0.680	**3.380**	0.044	0.024	**2.530**	**1.370**	**6.750**
	13–17.9	0.018	0.010	0.790	0.430	**2.110**	0.036	0.019	**1.580**	0.860	**4.220**
	18–34.9	0.014	0.008	0.670	0.360	**1.790**	0.029	0.015	**1.340**	0.720	**3.570**
	35–64.9	0.011	0.006	0.660	0.360	**1.770**	0.021	0.012	**1.330**	0.720	**3.550**
	≥65	0.010	0.006	0.760	0.410	**2.020**	0.021	0.011	**1.510**	0.820	**4.040**
Thai rice noodles	3–5.9	0.052	ND	**1.480**	ND	**8.990**	0.104	ND	**2.970**	ND	**17.970**
	6–12.9	0.036	ND	**1.020**	ND	**6.190**	0.071	ND	**2.040**	ND	**12.380**
	13–17.9	0.026	ND	0.640	ND	**3.870**	0.052	ND	**1.280**	ND	**7.740**
	18–34.9	0.025	ND	0.540	ND	**3.270**	0.050	ND	**1.080**	ND	**6.550**
	35–64.9	0.021	ND	0.540	ND	**3.250**	0.043	ND	**1.070**	ND	**6.510**
	≥65	0.013	ND	0.610	ND	**3.710**	0.027	ND	**1.220**	ND	**7.410**

HQ of exposure to Al from the consumption of egg noodles was not calculated because Al was not detected in any of the egg noodle samples. ND: not detected. PCTL: percentile. Bold figures indicate HQ values greater than 1. JECFA PTWI: provisional tolerable weekly intake of 2 mg/kg bw/week established by the Joint FAO/WHO Expert Committee on Food Additives. EFSA TWI: tolerable weekly intake of 1 mg/kg bw/week established by the European Food Safety Authority.

## Data Availability

The data used to support the findings of this study can be made available by the corresponding author upon request.
